# *Sine Systemate Chaos?* A Versatile Tool for Earthworm Taxonomy: Non-Destructive Imaging of Freshly Fixed and Museum Specimens Using Micro-Computed Tomography

**DOI:** 10.1371/journal.pone.0096617

**Published:** 2014-05-16

**Authors:** Rosa Fernández, Sebastian Kvist, Jennifer Lenihan, Gonzalo Giribet, Alexander Ziegler

**Affiliations:** 1 Museum of Comparative Zoology, Department of Organismic and Evolutionary Biology, Harvard University, Cambridge, Massachusetts, United States of America; 2 Ziegler Biosolutions, Waldshut-Tiengen, Germany; Imperial College London, United Kingdom

## Abstract

In spite of the high relevance of lumbricid earthworms (‘Oligochaeta’: Lumbricidae) for soil structure and functioning, the taxonomy of this group of terrestrial invertebrates remains in a quasi-chaotic state. Earthworm taxonomy traditionally relies on the interpretation of external and internal morphological characters, but the acquisition of these data is often hampered by tedious dissections or restricted access to valuable and rare museum specimens. The present state of affairs, in conjunction with the difficulty of establishing primary homologies for multiple morphological features, has led to an almost unrivaled instability in the taxonomy and systematics of certain earthworm groups, including Lumbricidae. As a potential remedy, we apply for the first time a non-destructive imaging technique to lumbricids and explore the future application of this approach to earthworm taxonomy. High-resolution micro-computed tomography (μCT) scanning of freshly fixed and museum specimens was carried out using two cosmopolitan species, *Aporrectodea caliginosa* and *A. trapezoides*. By combining two-dimensional and three-dimensional dataset visualization techniques, we demonstrate that the morphological features commonly used in earthworm taxonomy can now be analyzed without the need for dissection, whether freshly fixed or museum specimens collected more than 60 years ago are studied. Our analyses show that μCT in combination with soft tissue staining can be successfully applied to lumbricid earthworms. An extension of the approach to other families is poised to strengthen earthworm taxonomy by providing a versatile tool to resolve the taxonomic chaos currently present in this ecologically important, but taxonomically neglected group of terrestrial invertebrates.

## Introduction

In one of the first comprehensive investigations into earthworm (Clitellata: ‘Oligochaeta’) taxonomy, written more than a century ago, Michaelsen [1] featured the prophetic words *sine systemate chaos* in the title of his treatise. This seminal work, together with Stephenson's contribution to earthworm systematics published thirty years later [2], has constituted the backbone of the classical phylogenetic system of earthworms. However, in many higher earthworm taxa, the systems proposed by Michaelsen and Stephenson were based on a small number of key characters, which unfortunately failed to create a sound classification of the group. Since then, earthworm systematics has been gradually eroded by an ongoing controversy over how to classify various groups of these organisms. References to the instability of earthworm taxonomy are frequent in the literature (e.g., [3]), and studies have emphasized the lack of robust classifications on various taxonomic levels (e.g., ‘the family-level classification of the megascolecid earthworms is in chaos’ [4]).

In particular, this unfortunate state of affairs holds true for Lumbricidae Claus, 1876. The lumbricid system of classification has been referred to as an ‘unequalled chaos’ [5], [6]. Despite their biological, ecological and economic importance, the taxonomic status and evolutionary relationships of most lumbricid genera are still under debate. The lack of agreement concerning the ranking of diagnostic morphological characters in lumbricid earthworms has led to the creation of numerous synonyms at the species or genus level. For instance, the overlap of certain morphological features (e.g., shape of prostomium; position of chaetae; position and shape of sexual organs including clitellum, tubercula pubertatis, testes, ovaries, and spermathecae) has complicated species identification. Unfortunately, overlap in these key characters in closely- and distantly-related species is pervasive [6]–[13]. The instability of lumbricid earthworm taxonomy can be linked to three major issues: a high rate of synonymy, the establishment of ‘catch-all’ polyphyletic genera, and the existence of broad ‘species complexes’ [6]–[9], [14].

During the last years, newly developed techniques such as DNA-based systematics, DNA barcoding, or integrative taxonomy [15]–[17] have been used to complement the classical, strictly morphology-based taxonomy in earthworms. Earthworm identification requires detailed anatomical studies, which have traditionally been performed through specimen dissection [7]. Alas, earthworm dissections can be a time-consuming affair, require a high degree of expertise and, importantly, often cannot be applied to valuable and rare museum specimens. In order to increase the speed of advancement, taxonomists are in dire need of quick, reproducible, and non-destructive techniques that would permit the investigation of internal earthworm anatomy more reliably and on a larger scale.

A number of imaging techniques can be employed to successfully visualize internal structures of zoological specimens non-invasively, including minute samples. The list of methodologies comprises optical projection tomography [18], magnetic resonance imaging [19], synchrotron-radiation micro-computed tomography [20], [21], autofluorescence imaging [22], and micro-computed tomography [23]. Recent reviews provide a comprehensive overview of non-invasive imaging techniques and their application to terrestrial and marine organisms [24], [25]. Because of its high speed, low cost, ease of use, and the achievable high dataset resolution, micro-computed tomography (μCT) has recently evolved to become an important imaging tool in zoological studies. Based on the physical principle of X-ray imaging, μCT is particularly suitable for the visualization of mineralized tissues, and has thus been broadly applied in paleontology (e.g., [26], [27]). However, electron-dense contrast agents such as iodine, lead, tungsten, or osmium can be employed to visualize soft parts as well [28]–[34].

Using this approach, samples pertaining to a wide range of extant fauna have been successfully analyzed in the past, with the animals under study either being scanned in the form of isolated body parts or as whole, intact specimens. These taxa include Porifera [35], Chelicerata [36]–[39], Hexapoda [40]–[46], Bryozoa [47], Polychaeta [48], Mollusca [47], [49], [50], and Vertebrata [29]–[34], [51]–[57]. Preliminary analyses of a clitellate (*Allolobophora* sp.) using soft tissue staining techniques in combination with μCT scanning suggested that earthworms would be suitable organisms for this non-destructive imaging approach [58].

The present study represents the first attempt at comparative μCT scanning of earthworm specimens. Due to the fact that proper earthworm identification at the species level often relies on subtle differences, a correct interpretation of the resulting imagery is of particular importance for this group of animals. In order to test any potential limitations of this novel approach in earthworm taxonomy, specimens from two closely-related species, i.e., *Aporrectodea caliginosa* (Savigny, 1826) and *A. trapezoides* (Dugès, 1828), were chosen. Due to morphological and ecological similarities between these two congeners, the taxonomic status of the *A. caliginosa* species complex has been a matter of debate for more than a century. The morphological differences between the two species are very subtle and include the shape of the tubercula pubertatis on segments XXXI–XXXIII (in the form of two pairs of scale-like protuberances in *A. caliginosa* or in the form of a continuous pad-like organ in *A. trapezoides*) as well as the mode of reproduction (sexual in *A. caliginosa* or parthenogenetic in *A. trapezoides*). However, specimens with intermediate characteristics have been reported, rendering differentiation between the two species difficult. Although phylogenetic studies separate both species [59], [60], their taxonomic status has been in constant flux (e.g., [61]) and several studies have attempted, using both morphological and molecular tools, to resolve this taxonomic dilemma (summarized in [3]).

In order to provide a further tool for earthworm taxonomy, the present study aims to assess the usefulness of μCT scanning in combination with soft tissue staining to identify morphological characters of importance for earthworm taxonomy, and to compare the application of this non-destructive imaging approach to museum and freshly fixed specimens. Furthermore, the quality of μCT-derived data in comparison with results derived from traditional dissection techniques are discussed.

## Materials and Methods

### Specimens

Six specimens were used for μCT scanning: three freshly fixed specimens of *Aporrectodea caliginosa* collected in 2013 in Cambridge, Massachusetts, USA; a museum specimen collected in 1967 in Mérida, Venezuela; a freshly fixed specimen of *A. trapezoides* collected in 2013 in Menorca, Balearic Islands, Spain; and a museum specimen collected in 1945, in Oregon, USA. Freshly fixed specimens were collected either by digging 20–40 cm into soil or by surface collection following rainfall. The two species are not listed as endangered or protected, and no specific collecting permits were required for the four localities. All specimens were identified following the key provided in [3]. Table 1 gives an overview of the specimens employed for imaging, as well as GPS coordinates for some of the localities. All the freshly fixed and museum specimens that were scanned in this study are deposited in the collection of the Department of Invertebrate Zoology (IZ), Museum of Comparative Zoology (MCZ), Cambridge, Massachusetts, USA. Specimens information and associated images can be found on MCZbase (http://mczbase.mcz.harvard.edu).

**Table 1:** List of specimens used for μCT scanning.

Species	Specimen data	Specimen size	Staining parameters	Scanning parameters
*Aporrectodea caliginosa* (Savigny, 1826)	MCZ IZ 25152; collected March 10, 2013; Cambridge, Massachusetts, USA; GPS: 42.378658, 71.11616; fixed in 95% EtOH, stored in 95% EtOH	4.1 mm diameter, 6.4 cm length	10% I_2_KI for 4 days	65 kV, 100 µA, 50 min 4 s, 8.88 µm
	See above	See above	10% I_2_KI for 10 days	65 kV, 100 µA, 32 min 58 s, 8.88 µm
	MCZ IZ 25150; collected March 10, 2013; Cambridge, Massachusetts, USA; fixed in 95% EtOH, stored in 95% EtOH	3.6 mm diameter, 8.1 cm length	0.3% PTA for 4 days	70 kV, 100 µA, 48 min 40 s, 7.11 µm
	See above	See above	0.3% PTA for 9 days	65 kV, 100 µA, 32 min 26 s, 14.92 µm
	MCZ IZ 24805; collected March 10, 2013; Cambridge, Massachusetts, USA; fixed in 95% EtOH, stored in 95% EtOH	4.5 mm diameter, 6.1 cm length	0.3% PTA for 21 days	70 kV, 100 µA, 1 h 20 min 54 s, 9.95 µm
	MCZ IZ 95557; collected December 2, 1967; Mérida, Venezuela; fixed in formalin, stored in 70% EtOH	4.7 mm diameter, 5.4 cm length	0.3% PTA for 28 days	70 kV, 100 µA, 1 h 37 min 1 s, 13.15 µm
*Aporrectodea trapezoides* (Dugès, 1828)	MCZ IZ 24804; collected January 3, 2013; Menorca, Spain; GPS: 40.01056, 3.87817; fixed in 95% EtOH, stored in 95% EtOH	5.2 mm diameter, 8.0 cm length	0.3% PTA for 28 days	70 kV, 100 µA, 1 h 36 min 59 s, 8.17 µm
	MCZ IZ 95901; collected December 14, 1945; Oregon, USA; fixed in formalin, stored in 70% EtOH	4.5 mm diameter, 10.8 cm length	0.3% PTA for 28 days	70 kV, 100 µA, 1 h 37 min 5 s, 8.17 µm

Scanning parameters were: source voltage (kV), source current (µA), scanning time (h, min, s), and isotropic voxel resolution of the reconstructed 3D dataset (µm). The embedded hyperlinks provide direct access to the specimen entries in MCZbase (e.g., MCZ IZ 25152).

### Specimen staining

The six specimens intended for scanning were stained in 50 mL Falcon tubes (BD Biosciences, San Jose, California, USA) on a rocking table, using either a 10% iodine solution (Lugol's solution, iodine potassium iodide, I_2_KI) in ethanol (at 95% concentration) or a 0.3% phosphotungstic acid (PTA) solution, which also included 3% dimethyl sulfoxide (DMSO) to increase cell membrane permeability, in ethanol (at 95% concentration). Following an initial trial phase with different solutions as well as various lengths of staining and scanning times, specimens were finally stained for a total of three to four weeks using the PTA solution. Iodine staining was rejected due to the potentially significant specimen shrinkage that may occur at higher concentrations of I_2_KI [62]. Once stained with the PTA solution, specimens were placed in plastic drinking straws with 6 mm diameter, which were heat-sealed at the bottom, filled with the solution in which the worms had been kept during staining, and closed at the top end using Parafilm (Pechiney Plastic Packaging Co., Chicago, Illinois, USA). It is important to note that, typically, stained specimens are submerged in clean ethanol for scanning (e.g., [28], [47]) However, we noticed that performing the scan with the organisms still submerged in the PTA solution used for staining did not lead to negative results. We assume that most of the staining molecules must have been absorbed by the tissue. In order to increase the isotropic voxel resolution of the three-dimensional (3D) datasets, only the first ca. 35 segments of each specimen were scanned. These segments harbor all internal and external structures commonly used as diagnostic characters in earthworm taxonomy.

### Micro-computed tomography

Imaging was performed using a SkyScan 1173 μCT scanner (Bruker MicroCT, Kontich, Belgium) equipped with a Hamamatsu 130/300 tungsten X-ray source and a FlatPanel Sensor camera detector with 2240×2240 pixels. Scanning parameters were as follows: source voltage = 65–70 kV, source current = 100 µA, exposure time = 1,000 ms, frames averaged = 2–6, frames acquired over 180° = 960, filter = no, binning = no, flat field correction = activated, and scanning time = about 45–100 min. Reconstruction of the raw data was accomplished using the software provided with the scanner (NRecon 1.6.6.0, Bruker MicroCT, Kontich, Belgium). Various settings were employed to enhance image contrast and to compensate for ring and streak artifacts. These dataset reconstruction parameters were: smoothing = no, ring artifact correction = 5–11, and beam hardening correction = activated.

The four most representative μCT scans have been deposited in the voxel repository GigaDB [63]. In addition, an accompanying publication provides further information about data availability, quality, and requirements [64].

### Dissection and photography

In order to compare our μCT scanning results with traditional dissections commonly carried out by earthworm taxonomists, freshly fixed specimens identified as *A. caliginosa*, collected at the same time and location as the conspecific specimens used for μCT scanning, were dissected. These dissections were performed by making a dorsal incision to the body wall and exposing the internal organs. This destructive approach involves removal of a piece of integument to expose the internal organs, and partial or complete removal of some internal structures, such as the seminal vesicles or the septa. Auto-montage images of the dissected animals were acquired using a Leica MZ12.5 stereomicroscope (Leica Microsystems, Wetzlar, Germany) with an attached JVC KY-F75U digital imaging camera (JVC, Wayne, New Jersey, USA). Image stacks consisting of 17 separate photographs (exposure = 250 ms) were merged using the software Auto-Montage Pro 5.02.0096 (Syncroscopy, Frederick, Maryland, USA) with the following settings: method = fixed, optimize = precision, and patch size = 95.

### 3D visualization and modeling

The acquired 3D μCT datasets were analyzed using computer systems equipped with a 64-bit Windows operating system (Windows 7, Microsoft Co., Redmond, Washington, USA), a multi-core CPU, as well as a minimum of 6 GB of main and 1 GB of video RAM. Two-dimensional (2D) image slicing was accomplished with the free software DataViewer (http://www.skyscan.be/products/downloads.htm) and ImageJ 1.44p (http://imagej.nih.gov/ij/) using the plug-in Volume Viewer 2.01 (http://rsb.info.nih.gov/ij/plugins/volume-viewer.html). 3D rendering was performed by employing the commercial software Amira 5.2 (FEI Co., Hillsboro, Oregon, USA) as well as the free software Drishti (http://sf.anu.edu.au/Vizlab/drishti/index.shtml). The interactive 3D PDF model was created by manually segmenting, then surface-rendering and smoothing selected structures in Amira 5.2, assembling them in the commercial Adobe 3D Reviewer software (Adobe Systems, San Jose, California, USA), before finally exporting them as an interactive PDF file [65], [66].

## Results

The first section of the Results provides a description of structures identifiable in the μCT dataset of the freshly fixed specimen MCZ IZ 24805 (*Aporrectodea caliginosa*), which had been PTA-stained for four weeks. File S1 shows virtual 2D sections through the entire dataset. Following this first section, we present results concerning the morphological differences between the two *Aporrectodea* species, and a comparison of the performance of the approach on freshly fixed and museum material. In addition, we provide information on scanning artifacts encountered during the experiments. All acquired μCT datasets were analyzed qualitatively using 3D volume rendering techniques in combination with 2D virtual dataset slicing tools. In addition, a 3D PDF model, which permits interactive access to selected internal and external structures is available as File S2. The accompanying descriptive notes are provided as File S3.

### Identification of characters conventionally used in earthworm taxonomy

#### External features

**Figure 1:**
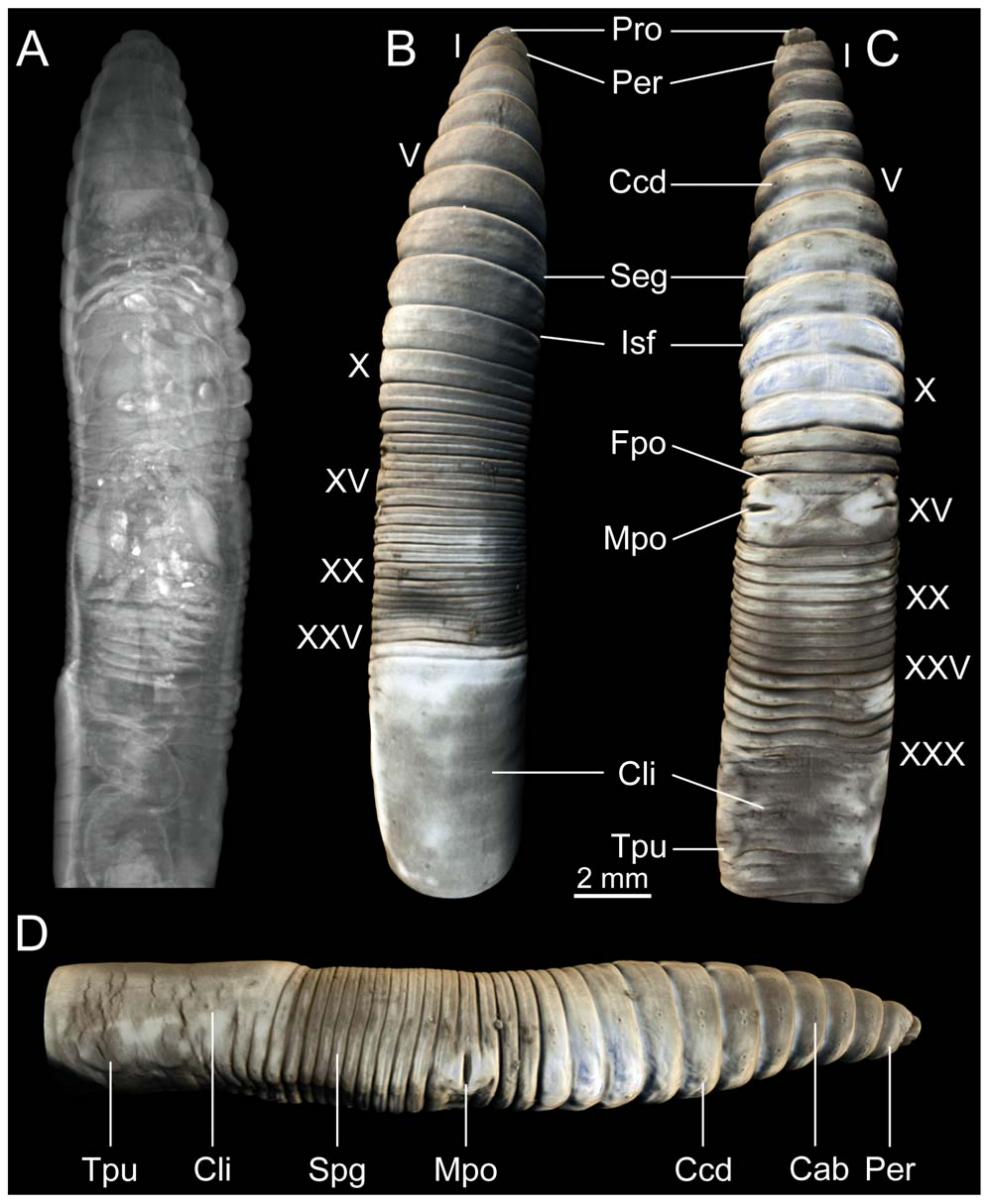
Visualization of the μCT dataset of a PTA-stained specimen of *Aporrectodea caliginosa* (MCZ IZ 24805, freshly fixed specimen). Anterior facing upwards (A–C) or to the right (D). (A) 2D X-ray projection image of the dataset. (B) Dorsal view of a false-color volume rendering of the dataset. (C) Ventral view. (D) Lateral view. Abbreviations: Cab, chaetae ab; Ccd, chaetae cd; Cli, clitellum; Fpo, female pore; Isf, intersegmental furrow; Mpo, male pore; Per, peristomium; Pro, prostomium; Seg, segment; Spg, sperm groove; Tpu, tubercula pupertatis. Roman numerals denote segment numbers.

**Figure 2:**
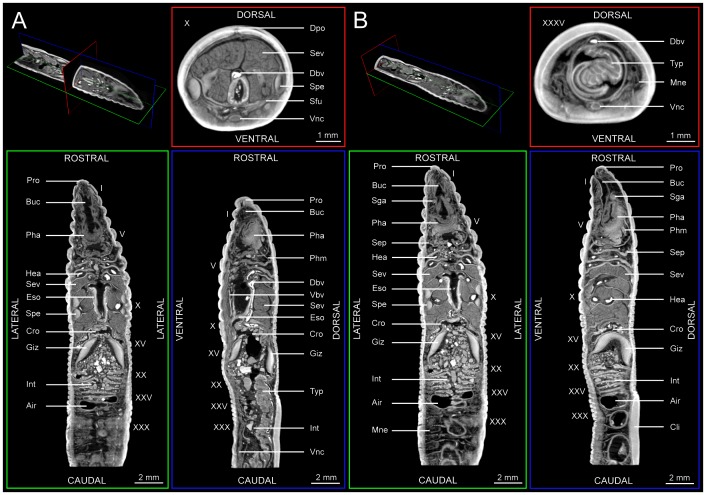
Selected virtual 2D sections through the μCT dataset of a PTA-stained specimen of *Aporrectodea caliginosa* (MCZ IZ 24805, freshly fixed specimen). (A) Transverse section at segment X (red), coronal section at the level of the buccal cavity (green), and sagittal section at the level of the typhlosole (blue). (B) Transverse section at segment XXXV (red), coronal section at the level of the spermathecae (green), and sagittal section at the level of the hearts (blue). Abbreviations: Air, trapped air; Buc, buccal cavity; Cli, clitellum; Cro, crop; Dbv, dorsal blood vessel; Dpo, dorsal pore; Eso, esophagus; Giz, gizzard; Hea, heart; Int, intestine; Mne, metanephridium; Pha, pharynx; Phm, pharyngeal muscle; Pro, prostomium; Sep, septum; Sev, seminal vesicle; Sga, supraesophageal ganglion; Sfu, sperm funnel; Spe, spermatheca; Typ, typhlosole; Vbv, Ventral blood vessel; Vnc, ventral nerve cord. Roman numerals denote segment numbers.

A classical 2D X-ray projection image of the anterior part of the PTA-stained, freshly fixed specimen of *A. caliginosa* reveals full penetration of the staining agent throughout all tissues (Fig. 1A). The general shape of the anterior body, its segments, as well as the intersegmental furrows can be readily identified in the μCT-based 3D volume renderings of the external surface (Fig. 1B–D). The prostomium, which surrounds the mouth, is epilobous and extends into a third of the first segment, the peristomium (Fig. 1B, C). The clitellum lies dorsally and begins to form at segment XXVII. The precise position of pores, often relevant for taxonomic identification, tends to be difficult to identify using traditional techniques. The μCT dataset allows unambiguous identification of most of these structures, in particular when 3D renderings (Fig. 1B–D) and virtual 2D sections (Fig. 2) are used in combination. On the dorsal side, the dorsal pores cannot be identified in the 3D renderings (Fig. 1B), but are visible in the 2D virtual sections (Fig. 2A). A number of features can be discerned on the ventral side (Fig. 1C), in particular the female (XIV) and male pores (XV). The latter constitute a large slit between chaetae bc and are surrounded by a glandular crescent protruding into the neighboring segments. Furthermore, the papillae of chaetae ab in segments IX, X, and XI can be identified. The nephridiopores are too small to be seen in the volume renderings, but they can be traced in the 2D sections. A lateral view (Fig. 1D) reveals the position of the four pairs of chaetae as well as the sperm groove. However, the spermathecal pores (present in intersegmental furrows IX–X and X–XI) can only be identified in the 2D virtual sections.

#### Coelomic cavity

Virtual 2D sections through different parts of the body reveal the general arrangement of the coelomic compartments (Fig. 2). Each segment is separated from the next by a septum. Septa in segments V–VI to IX–X are particularly muscular (especially septum VII–VIII), a feature common to some lumbricid taxa [67]–[69]. The mesenteries and the mesothelium are identifiable in the virtual 2D sections, in particular in the segments surrounding the intestine (from segment XX onwards). Chaetae ab (in tumescences in segments IX–XI) and chaetae cd (with typical lumbricine arrangement, i.e., eight chaetae per segment in pairs and in longitudinal series) are retracted, but can be observed particularly well in the 2D virtual sections. The chaetigerous sacs and muscles are in general difficult to discern, but those of the sexual chaetae can be identified in the virtual 2D sections.

#### Body wall

A transverse section through a given segment permits identification of parts of the body wall. Beginning from the outside, the cuticle is followed by the epidermis, which surrounds the circular and longitudinal muscle layers, and finally, the peritoneum, which borders the coelomic cavity. While the cuticle, the epidermis, and the two muscle layers can be differentiated in most sections (Fig. 2), the peritoneum is too thin to be discerned with certainty at the given dataset resolution (9.95 µm isotropic).

#### Digestive system

**Figure 3:**
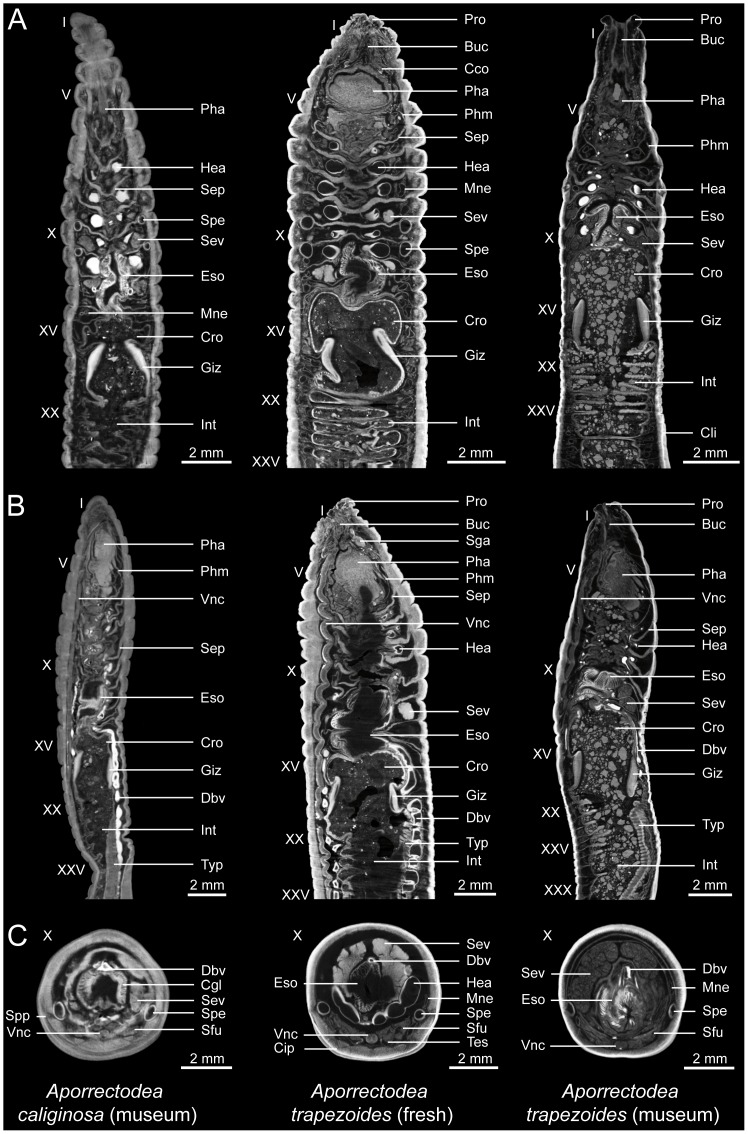
Selected virtual 2D sections through μCT datasets of PTA-stained specimens of *Aporrectodea caliginosa* (MCZ IZ 95557, museum specimen, left), *A. trapezoides* (MCZ IZ 24804, freshly fixed specimen, center), and *A. trapezoides* (MCZ IZ 95901, museum specimen, right). (A) Coronal view. (B) Sagittal view. (C) Transverse view. Abbreviations: Buc, buccal cavity; Cco, cerebral commissure; Cgl, calciferous gland; Cip, chaetal insertion point; Cli, clitellum; Cro, crop; Dbv, dorsal blood vessel; Eso, esophagus; Giz, gizzard; Hea, heart; Int, intestine; Mne, metanephridium; Pha, pharynx; Phm, pharyngeal muscle; Pro, prostomium; Sep, septum; Sev, seminal vesicle; Sfu, sperm funnel; Sga, supraesophageal ganglion; Spe, spermatheca; Spp, spermathecal pore; Tes, testes; Typ, typhlosole; Vnc, ventral nerve cord. Roman numerals denote segment numbers.

Most compartments of the digestive tract can be readily identified (Fig. 2). The course of the sediment as well as its composition throughout the digestive tract can be traced without difficulty – see File S4 for animated 3D volume renderings of the entire dataset. The anterior foregut is composed of the mouth (surrounded by the prostomium), the buccal cavity (I–III), a muscular pharynx (III–V) with dozens of pharyngeal muscles that can be traced all the way to their attachment sites on the dorsal and lateral body wall, and a thickened, muscular esophagus (VI–XI). The calciferous gland is characterized by the presence of two well-developed pouches with multiple layers in segment X (Fig. 3C) and the absence of lateral enlargements in segment XIII. The posterior foregut is composed of a wide crop (XV–XVI), followed by a muscular gizzard (XVII–XVIII). This latter structure terminates in a pre-intestinal valve. Following the foregut, which is entirely lined by cuticle, the midgut is composed of the intestine (starting at segment XIX). The intestinal walls are thinner than those of most parts of the foregut. A particularly saccular part of the intestine with numerous transverse folds can be found in the segments directly following the gizzard (XIX–XXVI). A prominent spade-shaped typhlosole starts to form in segment XXII. The elements of the hindgut (i.e., rectum and anus) are outside the field of view of the present dataset.

#### Vascular system

The intense staining of hemoglobin inside the vascular system facilitates the detection of blood vessels. They appear as clearly contoured dilations with a bright filling (Figs. 2, 3). The dorsal blood vessel can be traced through almost the entire length of the dataset. The ventral blood vessel can equally be traced all the way to the end of the field of view. In segments VI–XI, paired lateral hearts of varying sizes connect the dorsal blood vessel to its ventral counterpart. In the posterior part of the dataset, dorsoparietal vessels join the dorsal blood vessel with the small subneural vessel. The lateral neural and the ventroparietal vessels, as well as the dorsointestinal and ventrointestinal vessels can be identified in some of the virtual 2D sections, but the given resolution of the dataset (or the lack of hemoglobin in these parts of the vessels) somewhat limit their visibility.

#### Excretory system

Throughout most segments, metanephridia can be observed. These structures can be identified as structures with J-shaped bladders (Fig. 2B) that are hooking caudally. The nephrostome is too small to be clearly visualized at the given dataset resolution, while the nephridiopores are visible.

#### Reproductive system

Two pairs of sperm-filled spermathecae can be observed in intersegments IX–X and X–XI (Fig. 2). Their pores open in-between segments IX and X as well as segments X and XI. The testes are barely visible, but their sperm funnels are clearly shown. From these, a pair of vasa deferentia leading to the male pores is seen in segment XV. The massive, paired seminal vesicles can be seen in segments IX to XII, occupying the majority of the coelomic cavity. Paired metagynous ovaries in segment XIII and paired ovisacs in segment XIV (both containing several eggs) can be identified and are closely associated with the oviducts, which in turn lead to the female pores of segment XIV.

#### Nervous system

The brain is composed of paired supraesophageal (or cerebral) ganglia that lie dorsal to the buccal cavity in segment III (Fig. 2). The paired cerebral commissure connects the supraesophageal ganglia through segment IV with the paired subesophageal ganglia lying in segment V. The large ventral nerve cord starts to form in segment VI. The segmental nerves emanating from the ventral nerve cord can be seen in some sections, but never extending further than chaetae cd. The finer nerves are too small to be seen at the given dataset resolution.

### *Aporrectodea caliginosa* or *Aporrectodea trapezoides*? Differences between the two analyzed species

Two pairs of sperm-filled spermathecae can be observed in intersegments IX–X and X–XI of the freshly fixed specimen of *A. caliginosa* (Fig. 2), while only empty spermathecae can be observed in the two specimens of *A. trapezoides* as well as the museum specimen of *A. caliginosa* (Fig. 3). In addition, not only the spermathecae, but also the seminal vesicles are much smaller in these three latter specimens compared to the freshly fixed individual of *A. caliginosa*. In the freshly fixed specimen of *A. caliginosa*, spermathecae and seminal vesicles occupy a large part of the coelomic cavity in segments IX–XII. While the presence of empty spermathecae may not be an indicator for parthenogenetic reproduction, several anatomical differences seem to indicate that the museum specimen labeled as *A. caliginosa* (MCZ IZ 95557) could be a parthenogenetic individual. However, because *A. caliginosa* is a sexual species (no parthenogenetic specimens have ever been described), this specimen could be a case of misidentification, and the sample may actually be *A. trapezoides* (or any senior synonym in use at the time of identification).

Ultimately, the solution for the problematic taxonomy of these two extremely similar species will rest on the inspection of the topotype of *A. caliginosa*, which is presumably located at the Muséum National d'Histoire Naturelle in Paris, France, and on the designation of a neotype of *A. trapezoides* from the possible type locality of this species (i.e., Montpellier, France) [3].

### Comparison of the performance of μCT scanning between freshly fixed and museum specimens

In terms of the efficacy of the staining procedure and the resulting image quality, we found no significant differences between freshly fixed specimens and those stored in formaldehyde or ethanol for extended periods of time. Similarly, we did not find any differences in the performance of the approach between the two species used in the present study. The only factor that seems to affect the method chosen here is body size. Due to the relatively slow rate of diffusion of the staining agent, any increase in specimen width will result in a longer staining time. Our initial trial phase with different durations of staining led to insufficient penetration of specimens stained for about a week. This problem was successfully solved by staining all specimens for longer periods of time (i.e., three to four weeks).

The only notable difference between freshly fixed and museum specimens was that, depending on the fixation protocol originally used, some museum specimens were more contracted than the freshly fixed ones, resulting in occasional differences in organ size and shape. Furthermore, we found that it is of importance to properly arrange the animals in a standardized orientation prior to scanning. Therefore, all specimens were scanned following dorso-ventral and rostro-caudal straightening inside plastic straws. This position closely resembles the orientation of earthworms during dissection, facilitating a comparison of results derived from both techniques.

### Artifacts

**Figure 4:**
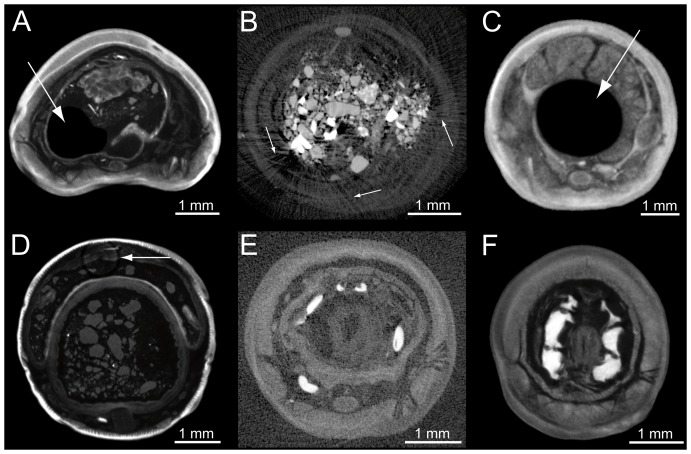
Artifacts encountered in the μCT datasets of the six earthworm specimens analyzed in the course of this study. (A) The arrow points to a large void inside the digestive tract caused by an air bubble trapped within the gut. (B) The arrows denote streak artifacts caused by highly electron-dense sediment particles located inside the digestive tract. (C) The arrow points to a circular void at the specimen's center caused by insufficient staining of the sample. (D) The arrow points to a ring artifact caused by faulty detector pixels. (E) Insufficient contrast between stained tissue and the surrounding solution due to stain leakage into the surrounding liquid. (F) Blurred image and consequently reduced voxel resolution, caused by specimen movement during scanning, leading to a reduced isotropic voxel resolution.

Artifacts related to the specimen's biology, the staining procedure, or the scanning methodology may affect dataset quality. Pockets of air (Fig. 4A) were found inside the digestive tract of most specimens (Figs. 2A, B; 3A, B). These air pockets appear as black voids, because the staining solution does not diffuse into them. However, these artifacts are easily identified, do not affect volume rendering, and do not compromise the integrity of the surrounding anatomical features. Another problem encountered was the presence of pronounced streak artifacts caused by electron-dense sediment particles with higher X-ray attenuation than that of the surrounding tissues (Fig. 4B).

Furthermore, imperfect staining was a prominent artifact seen in all specimens treated with short staining times (Fig. 4C). This artifact is caused by the relatively slow molecular diffusion rate of the staining solution into the specimen. The almost circular void at the core of the animal shows that the size of the staining molecule and animal width are the main properties governing the transfer rate of the staining solution into the specimen. The thickest specimen analyzed (5.2 mm diameter, center specimen in Fig. 3) showed imperfect staining, despite four weeks of consecutive immersion in the staining solution. Based on the six specimens scanned, a diffusion rate of approximately 1 mm per week was estimated for the PTA solution used.

Another type of artifact frequently observed was the presence of concentric rings (Fig. 4D), which are caused by faulty detector elements on the pixel detector array. Algorithms can be employed to remove most ring artifacts during dataset reconstruction. However, an excessive presence of staining molecules within the liquid surrounding the specimen during scanning cannot be compensated for (Fig. 4E). Finally, movement of specimens during scanning resulted in datasets with blurred imagery and consequently a reduced isotropic voxel resolution (Fig. 4F).

## Discussion

**Figure 5:**
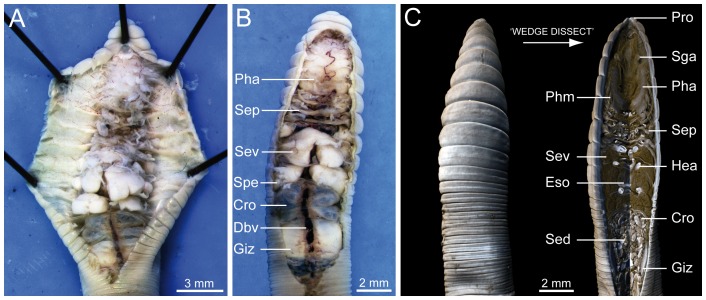
Comparison of traditional dissection techniques with a virtual dissection based on a μCT dataset of the earthworm species *Aporrectodea caliginosa* (MCZ IZ 24805, freshly fixed specimen). Specimens shown in dorsal view, with the anterior pointing upwards. (A) Traditional dissection technique based on a longitudinal cut of the dorsal body wall and vertical slitting of individual septa. (B) Traditional dissection technique based on the partial removal of the dorsal body wall, exposing the underlying organs, but leaving the lateral attachments of the septa intact. (C) False-color volume rendering of a μCT dataset (left) and virtual dissection of the same dataset (right) using the ‘wedge dissect’ function in the software Drishti (right). This virtual dissection simulates a longitudinal cut through the body wall and some of the underlying organs. Abbreviations: Cro, crop; Dbv, dorsal blood vessel; Eso, esophagous; Giz, gizzard; Hea, heart; Pha, pharynx; Phm, pharyngeal muscle; Pro, prostomium; Sed, sediment; Sep, septum; Sev, seminal vesicle; Sga, supraesophageal ganglion; Spe, spermatheca.

The present study is the first to explore the systematic application of a non-destructive imaging technique to earthworm specimens. One of the main advantages of the use of modern imaging techniques is that the acquired data are digital in nature and can thus be employed for numerous advanced visualization methods that would have been impossible using non-digital approaches. An example is provided in Figure 5, which shows a direct comparison of results derived from traditional earthworm dissection techniques (Fig. 5A, B) with a virtual dissection of a μCT dataset (Fig. 5C). One of the most conspicuous benefits of the virtual dissection is the possibility of observing internal organs in their natural anatomical context, which is often not the case when traditional dissection techniques are employed. Moreover, the specimens can be visualized and rotated in 3D, allowing for a better understanding of their complex morphology.

Furthermore, the present study demonstrates that the time span between initial specimen fixation and scanning does not represent a limiting factor for the performance of the technique. However, the fixation event itself is of importance, because variations in organ size and shape due to shrinkage or inflation may have occurred. Nonetheless, this effect would certainly also influence morphological results derived from traditional dissections of such specimens. Differences in the performance of the approach when applied to separate earthworm species can also be discarded, because staining properties of the same earthworm organs are likely to be similar, regardless of the species. However, one of the potential limitations of the application of μCT scanning in combination with soft tissue staining is the presence of artifacts (Fig. 4).

With regard to artifacts, the size of the specimen is the most important criterion, because the relatively slow diffusion rate of the various chemical agents used to stain biological tissues [28], [30], [31], [47], [70]–[72] can reduce the applicability of this approach to larger specimens. However, given the relatively small diameters of most earthworm species (in the millimeters to few centimeters range), this group of invertebrates still constitutes a suitable candidate for the approach. Due to the rapidity of the method once specimens have been stained and the relative ease of data acquisition, multiple specimens per species or sample should ideally be scanned in order to minimize the risk of obtaining data from a single, unsuitable specimen. However, any possible long-term effects on the electron density of soft tissues by different fixation protocols and specimen storage in formalin or ethanol have not yet been systematically analyzed and might have an effect on imaging results.

A factor with a significant impact on dataset quality is the achievable voxel resolution, as this parameter governs visibility of specific structures. Modern μCT systems can generate datasets with isotropic voxel resolutions in the single-digit-micrometer or even the nanometer scale. The scanner used in the present study was technically limited to ca. 7 µm spatial resolution, in turn meaning that our datasets ranging from about 7–15 µm isotropic voxel resolution more or less constitute the maximum resolution achievable given the size of the samples and the selected exposure times. However, different scanning systems or the application of synchrotron-based μCT [20], [21] can result in considerably higher spatial dataset resolution, opening up further opportunities to analyze minute earthworm species or juvenile specimens non-destructively in 3D.

Although the term ‘non-invasive’ has been employed in the context of this study, the staining of earthworm specimens should be considered as an invasive technique. In particular so, because the staining solutions cannot be fully washed out from the sample after scanning. In contrast, the exposure of museum specimens to the high X-ray dosages conventionally exerted during μCT experiments seems to be of lesser concern—a recent study suggests no detrimental effect to DNA molecules present in preserved museum specimens [73].

Due to the prevailing difficulties in delimiting lumbricid earthworm species, the broad application of μCT scanning to this group of terrestrial invertebrates could be particularly relevant, especially for future studies aimed at resolving the current state of lumbricid taxonomy. One of the novel methodological aspects presented in our study is the successful application of the technique to museum specimens fixed decades ago (Fig. 3). Museum collections around the world constitute a significant resource for taxonomic and systematic research and could now be systematically harvested using high-throughput non-invasive imaging techniques [74]. In this context, a ‘cybertaxonomy’ approach [48] could prove to be useful in providing the earthworm research community with reference specimens that have been verified online and agreed upon by several experts. Furthermore, because earthworm identification relies on several sexual characters, the identification of juvenile specimens is often inaccurate. An additional advantage of the μCT scanning approach is that it can be applied to hard-to-dissect juvenile specimens, which would facilitate species identification in taxa with unique somatic organs.

It is our conviction that μCT scanning represents a time- and cost-effective alternative to traditional dissection or in some cases even histological techniques. The cost of a μCT scan will certainly vary, depending on access to scanner hardware, reconstruction software, visualization equipment, and chemical reagents. However, μCT scanners are no longer limited to industrial, medical, or pharmaceutical research—in our particular case, for example, the scanner did not entail any associated user cost, and scanners are becoming a standard tool available in many research institutions. In addition, staining agents such as PTA or iodine are relatively cheap, while powerful, user-friendly data processing software are available even for free (see [25]). Multiple institutions with a focus on zoological studies now offer access to μCT systems, thus demonstrating the wide availability of this methodology. In this context, μCT scanning in combination with soft tissue staining constitutes a versatile tool for multidisciplinary approaches including morphological, ecological, and molecular methods that aim to assess the validity of characters of taxonomic relevance in earthworms.

## Conclusions

Earthworm taxonomy is in dire need of tools that can be used to provide further insights into the details of animal anatomy and morphology and that allow for the non-destructive analysis of type material or other valuable and rare museum specimens. Our study shows that μCT in combination with soft tissue staining can be successfully applied to earthworm taxa—whether freshly fixed or museum specimens are used—in order to investigate internal and external features that would otherwise be impossible to determine without dissection. At the isotropic voxel resolutions that were achieved, we were able to identify numerous internal structures such as spermathecae, seminal vesicles, metanephridia, ganglia, and sperm funnels, as well as features of the vascular and digestive systems. In addition, external features that may be difficult to identify through traditional dissection techniques (e.g., dorsal pores, nephridiopores, or pores of the spermathecae) are clearly discernible in the datasets. The protocols used in the present study can be employed to produce high-resolution imagery of hundreds of specimens in a much shorter timeframe than traditional protocols would permit. Moreover, the approach can likely be applied to various other taxa with similar anatomical characteristics, such as Hirudinida, Onychophora, Pentastomida, Sipuncula, Echiura, Priapulida, or Phoronida. The future advancement of earthworm taxonomy will likely come to rely upon non-invasive imaging techniques that allow rapid generation of massive amounts of digital data—an approach that is already commonplace in several other biological disciplines.

## Supporting Information

File S1 **Video file showing the full image stack of the μCT-scanned, PTA-stained specimen of** ***Aporrectodea caliginosa*** **(MCZ IZ 24805, freshly fixed specimen).**
(PDF)

File S2 **Interactive 3D PDF model of selected earthworm structures.** The 3D model is based on a μCT-scanned, PTA-stained specimen of *Aporrectodea caliginosa* (MCZ IZ 24805, freshly fixed specimen). Left-click to activate the embedded multimedia content (requires the use of Adobe Reader 9 or higher on Windows, Mac, and Linux systems). Use the ‘+/− zoom’ or ‘toggle full-screen’ options in order to maximize window size. Various pre-saved views can be accessed through the menu inside the viewer window or by opening the model hierarchy using the model tree icon. Grey, body wall; pink, digestive tract; red, circulatory system; brown, pharyngeal musculature; yellow, nervous system; orange, metanephridium; green, muscular septum; cyan, seminal vesicle; blue, spermatheca.
(PDF)

File S3 **Text file providing a step-by-step walkthrough of the pre-saved views shown in the interactive 3D PDF model (File S2).** The views mentioned in this description can be accessed through the menu inside the viewer window or by opening the model hierarchy using the model tree icon.
(PDF)

File S4 **Video file showing a virtual dissection of the anterior part of a μCT-scanned, PTA-stained specimen of** ***Aporrectodea caliginosa*** **(MCZ IZ 24805, freshly fixed specimen).** The video presents volume-rendered, false-colored coronal, sagittal, and transverse 3D views through the body of the specimen. Left-click to activate the embedded multimedia content (requires the use of Adobe Reader 9 or higher on Windows, Mac, and Linux systems).
(PDF)

## References

[1] Michaelsen W (1900) Oligochaeta; In: Sohn F, editor. Das Tierreich, 10. Lieferung, Vermes. Berlin: R. Friedländer und Sohn. 575 pp.

[2] Stephenson J (1930) The Oligochaeta; Press C, editor. Oxford: Clarendon Press. 978 pp.

[3] Blakemore R (2006) Cosmopolitan earthworms - an eco-taxonomic guide to the peregrine species of the world. Canberra: VermEcology. CD-ROM.

[4] Fender WM, McKey-Fender D (1990) Oligochaeta: Megascolecidae and other earthworms from western North America. In: Dindal DL, editor. Soil Biology Guide. New York: John Wiley and Sons. pp. 357–378.

[5] Ljungström PO (1972) Introduced earthworms of South Africa. On their taxonomy, distribution, history of introduction and on the extermination of endemic earthworms. Zoologische Jahrbücher - Abteilung für Systematik, Geographie und Biologie der Tiere 99: 1–181.

[6] Briones MJI, Morán P, Posada D (2009) Are the sexual, somatic and genetic characters enough to solve nomenclatural problems in lumbricid taxonomy? Soil Biology & Biochemistry 41: 2257–2271.

[7] Gates GE (1972) Burmese earthworms - introduction to systematics and biology of megadrile oligochaetes with special reference to southeast Asia. Transactions of the American Philosophical Society 62: 5–324.

[8] Pop AA, Wink M, Pop VV (2003) Use of 18S, 16S rDNA and cytochrome c oxidase sequences in earthworm taxonomy (Oligochaeta, Lumbricidae). Pedobiologia 47: 428–433.

[9] Bouché MB (1972) Lombriciens de France: écologie et systématique. Paris: Institut National de la Recherche Agronomique. 671 pp.

[10] Lavelle P, Spain AV (2001) Soil ecology. London: Kluwer Academic Publishers. 654 pp.

[11] Lee KE (1985) Earthworms: their ecology and relationships with soils and land use. Sydney: Academic Press. 411 pp.

[12] Edwards CA (2004) Earthworm ecology. Boca Ratón: CRC Press. 448 pp.

[13] Edwards CA, Bohlen PJ (1996) Biology and ecology of earthworms. London: Chapman and Hall. 426 pp.

[14] James SW, Porco D, Decaëns T, Richard B, Rougerie R, et al (2010) DNA barcoding reveals cryptic diversity in *Lumbricus terrestris* L., 1758 (Clitellata): resurrection of *L. herculeus* (Savigny, 1826). PLoS ONE 5: e15629. 21206917 10.1371/journal.pone.0015629 PMC3012069

[15] Hebert PDN, Ratnasingham S, deWaard JR (2003) Barcoding animal life: cytochrome c oxidase subunit 1 divergences among closely related species. Proceedings of the Royal Society B - Biological Sciences 270: S96–S99. 12952648 10.1098/rsbl.2003.0025 PMC1698023

[16] Dayrat B (2005) Towards integrative taxonomy. Biological Journal of the Linnean Society 85: 407–415.

[17] Kvist S (2013) Barcoding in the dark?: a critical view of the sufficiency of zoological DNA barcoding databases and a plea for broader integration of taxonomic knowledge. Molecular Phylogenetics and Evolution 69: 39–45. 23721749 10.1016/j.ympev.2013.05.012

[18] Sharpe J (2004) Optical projection tomography. Annual Review of Biomedical Engineering 6: 209–228. 10.1146/annurev.bioeng.6.040803.140210 15255768

[19] Ziegler A, Kunth M, Mueller S, Bock C, Pohmann R, et al (2011) Application of magnetic resonance imaging in zoology. Zoomorphology 130: 227–254.

[20] Betz O, Wegst U, Weide D, Heethoff M, Helfen L, et al (2007) Imaging applications of synchrotron X-ray phase-contrast microtomography in biological morphology and biomaterials science. 1. General aspects of the technique and its advantages in the analysis of millimetre-sized arthropod structure. Journal of Microscopy 227: 51–71. 17635659 10.1111/j.1365-2818.2007.01785.x

[21] Westneat MW, Socha JJ, Lee WK (2008) Advances in biological structure, function, and physiology using synchrotron x-ray imaging. Annual Review of Physiology 70: 119–142. 10.1146/annurev.physiol.70.113006.100434 18271748

[22] Haug JT, Haug C, Kutschera V, Mayer G, Maas A, et al (2011) Autofluorescence imaging, an excellent tool for comparative morphology. Journal of Microscopy 244: 259–272. 21883208 10.1111/j.1365-2818.2011.03534.x

[23] Stauber M, Müller R (2008) Micro-computed tomography: a method for the non-destructuve evaluation of the three-dimensional structure of biological specimens. Methods in Molecular Biology 455: 273–292. 18463825 10.1007/978-1-59745-104-8_19

[24] Boistel R, Swoger J, Krzic U, Fernández V, Gillet B, et al (2011) The future of three-dimensional microscopic imaging in marine biology. Marine Ecology 32: 438–452.

[25] Walter T, Shattuck DW, Baldock R, Bastin ME, Carpenter AE, et al (2010) Visualization of image data from cells to organisms. Nature Methods 7: S26–S41. 20195255 10.1038/nmeth.1431 PMC3650473

[26] Garwood R, Dunlop JA, Sutton MD (2009) High-fidelity X-ray microtomography reconstruction of siderite-hosted Carboniferous arachnids. Biology Letters 5: 841–844. 19656861 10.1098/rsbl.2009.0464 PMC2828000

[27] Lautenschlager S (2013) Cranial myology and bite force performance of *Erlikosaurus andrewsi*: a novel approach for digital muscle reconstructions. Journal of Anatomy 222: 260–272. 23061752 10.1111/joa.12000 PMC3632231

[28] Metscher BD (2009) MicroCT for developmental biology: a versatile tool for high-contrast 3D imaging at histological resolutions. Developmental Dynamics 238: 632–640. 19235724 10.1002/dvdy.21857

[29] Degenhardt K, Wright AC, Horng D, Padmanabhan A, Epstein JA (2010) Rapid 3D phenotyping of cardiovascular development in mouse embryos by micro-CT with iodine staining. Circulation: Cardiovascular Imaging 3: 314–322. 20190279 10.1161/CIRCIMAGING.109.918482 PMC3059892

[30] Jeffery NS, Stephenson RS, Gallagher JA, Jarvis JC, Cox PG (2011) Micro-computed tomography with iodine staining resolves the arrangement of muscle fibres. Journal of Biomechanics 44: 189–192. 20846653 10.1016/j.jbiomech.2010.08.027

[31] Tahara R, Larsson HCE (2013) Quantitative analysis of microscopic X-ray computed tomography imaging: Japanese quail embryonic soft tissues with iodine staining. Journal of Anatomy 223: 297–310. 23869493 10.1111/joa.12081 PMC3972050

[32] Baverstock H, Jeffery NS, Cobb SN (2013) The morphology of the mouse masticatory musculature. Journal of Anatomy 223: 46–60. 23692055 10.1111/joa.12059 PMC4487762

[33] Johnson JT, Hansen MS, Wu I, Healy LJ, Johnson CR, et al (2006) Virtual histology of transgenic mouse embryos for high-throughput phenotyping. PLoS Genetics 2: e61. 16683035 10.1371/journal.pgen.0020061 PMC1449902

[34] Prajapati SI, Rodríguez DR, Keller C (2014) Microscopic computed tomography-based virtual histology in embryos. In: Lewandoski M, editor. Mouse molecular embryology methods in molecular biology. New York: Springer pp. 291–296. 10.1007/978-1-60327-292-6_19 24318828

[35] Nickel M, Donath T, Schweikert M, Beckmann F (2006) Functional morphology of *Tethya* species (Porifera): 1. Quantitative 3D-analysis of *Tethya wilhelma* by synchrotron radiation based X-ray microtomography. Zoomorphology 125: 209–223.

[36] Klussmann-Fricke BJ, Prendini L, Wirkner CS (2012) Evolutionary morphology of the hemolymph vascular system in scorpions: a character analysis. Arthropod Structure & Development 41: 545–560. 22735399 10.1016/j.asd.2012.06.002

[37] Michalik P, Piacentini L, Lipke E, Ramírez MJ (2013) The enigmatic Otway odd-clawed spider (*Progradungula otwayensis* Milledge, 1997, Gradungulidae, Araneae): natural history, first description of the female and micro-computed tomography of the male palpal organ. ZooKeys 335: 101–112. 10.3897/zookeys.335.6030 PMC3800794 24146568

[38] Vöcking O, Uhl G, Michalik P (2013) Sperm dynamics in spiders (Araneae): ultrastructural analysis of the sperm activation p in the garden spider *Argiope bruennichi* (Scopoli, 1772). PLoS ONE 8: e72660. 24039790 10.1371/journal.pone.0072660 PMC3765205

[39] Kamenz C, Weidemann G (2009) Heavy metal - a contrasting substance for micro-tomographical visualization of scorpion book lungs. Micron 40: 911–917. 19520581 10.1016/j.micron.2009.05.007

[40] Bauder JAS, Handschuh S, Metscher BD, Krenn HW (2013) Functional morphology of the feeding apparatus and evolution of proboscis length in metalmark butterflies (Lepidoptera: Riodinidae). Biological Journal of the Linnean Society 110: 291–304. 24839308 10.1111/bij.12134 PMC4021108

[41] Eberhard MJB, Lang D, Metscher B, Pass G, Picker MD, et al (2010) Structure and sensory physiology of the leg scolopidial organs in Mantophasmatodea and their role in vibrational communication. Arthropod Structure & Development 39: 230–241. 20149895 10.1016/j.asd.2010.02.002

[42] Ribi W, Senden TJ, Sakellariou A, Limaye A, Zhang S (2008) Imaging honey bee brain anatomy with micro-X-ray-computed tomography. Journal of Neuroscience Methods 171: 93–97. 18400304 10.1016/j.jneumeth.2008.02.010

[43] Richards CS, Simonsen TJ, Abel RL, Hall MJR, Schwyn DA, et al (2012) Virtual forensic entomology: improving estimates of minimum post-mortem interval with 3D micro-computed tomography. Forensic Science International 220: 251–264. 22497703 10.1016/j.forsciint.2012.03.012

[44] Wilhelm G, Handschuh S, Plant J, Nemeschkal HL (2011) Sexual dimorphism in head structures of the weevil *Rhopalapion longirostre* (Olivier, 1807) (Coleoptera: Curculionoidea): a response to ecological demands of egg deposition. Biological Journal of the Linnean Society 104: 642–660.

[45] Zimmermann D, Randolf S, Metscher BD, Aspöck U (2011) The function and phylogenetic implications of the tentorium in adult Neuroptera (Insecta). Arthropod Structure & Development 40: 571–582. 21978822 10.1016/j.asd.2011.06.003

[46] Wilhelmi AP, Krenn HW (2012) Elongated mouthparts of nectar-feeding Meloidae (Coleoptera). Zoomorphology 131: 325–337.

[47] Metscher BD (2009) MicroCT for comparative morphology: simple staining methods allow high-contrast 3D imaging of diverse non-mineralized animal tissues. BMC Physiology 9: 11. 19545439 10.1186/1472-6793-9-11 PMC2717911

[48] Faulwetter S, Vasileiadou A, Kouratoras M, Dailianis T, Arvanitidis C (2013) Micro-computed tomography: introducing new dimensions to taxonomy. ZooKeys 263: 1–45. 10.3897/zookeys.263.4261 PMC3591762 23653515

[49] Golding RE, Ponder WF, Byrne M (2009) Three-dimensional reconstruction of the odontophoral cartilages of Caenogastropoda (Mollusca: Gastropoda) using micro-CT: morphology and phylogenetic significance. Journal of Morphology 270: 558–587. 19107810 10.1002/jmor.10699

[50] Kerbl A, Handschuh S, Nodl MT, Metscher B, Walzl M, et al (2013) Micro-CT in cephalopod research: investigating the internal anatomy of a sepiolid squid using a non-destructive technique with special focus on the ganglionic system. Journal of Experimental Marine Biology and Ecology 447: 140–148.

[51] Aslanidi OV, Nikolaidou T, Zhao JC, Smaill BH, Gilbert SH, et al (2013) Application of micro-computed tomography with iodine staining to cardiac imaging, segmentation, and computational model development. IEEE Transactions on Medical Imaging 32: 8–17. 22829390 10.1109/TMI.2012.2209183 PMC3493467

[52] Cox PG, Jeffery N (2011) Reviewing the morphology of the jaw-closing musculature in squirrels, rats, and Guinea pigs with contrast-enhanced microCT. Anatomical Record - Advances in Integrative Anatomy and Evolutionary Biology 294: 1612–1612. 10.1002/ar.21381 21538924

[53] Gignac PM, Kley NJ (2014) Iodine-enhanced microCT imaging: methodological refinements for the study of the soft-tissue anatomy of post-embryonic vertebrates. Journal of Experimental Zoology Part B: Molecular and Developmental Evolution 322: 166–176. 10.1002/jez.b.22561 24482316

[54] Holliday CM, Tsai HP, Skiljan RJ, George ID, Pathan S (2013) A 3D interactive model and atlas of the jaw musculature of *Alligator mississippiensis* . PLoS One 8: e62806. 23762228 10.1371/journal.pone.0062806 PMC3676386

[55] Lautenschlager S, Bright JA, Rayfield EJ (2013) Digital dissection - using contrast-enhanced computed tomography scanning to elucidate hard- and soft-tissue anatomy in the Common Buzzard *Buteo buteo* . Journal of Anatomy 224: 412–431. 24350638 10.1111/joa.12153 PMC4098676

[56] Saito S, Murase K (2012) *Ex vivo* imaging of mouse brain using micro-CT with non-ionic iodinated contrast agent: a comparison with myelin staining. British Journal of Radiology 85: E973–E978. 22674712 10.1259/bjr/13040401 PMC3500820

[57] Tsai HP, Holliday CM (2011) Ontogeny of the *Alligator* cartilago transiliens and its significance for sauropsid jaw muscle evolution. PLoS ONE 6: e24935. 21949795 10.1371/journal.pone.0024935 PMC3174982

[58] Ziegler A, Menze BH (2013) Accelerated acquisition, visualization, and analysis of zoo-anatomical data. In: Zander J, Mosterman JP, editors. Computation for Humanity. Boca Ratón: CRC Press. pp. 233–261.

[59] Fernández R, Almodóvar A, Novo M, Simancas B, Cosín DJD (2012) Adding complexity to the complex: new insights into the phylogeny, diversification and origin of parthenogenesis in the *Aporrectodea caliginosa* species complex (Oligochaeta, Lumbricidae). Molecular Phylogenetics and Evolution 64: 368–379. 22542691 10.1016/j.ympev.2012.04.011

[60] Pérez-Losada M, Ricoy M, Marshall JC, Domínguez J (2009) Phylogenetic assessment of the earthworm *Aporrectodea caliginosa* species complex (Oligochaeta: Lumbricidae) based on mitochondrial and nuclear DNA sequences. Molecular Phylogenetics and Evolution 52: 293–302. 19364539 10.1016/j.ympev.2009.04.003

[61] Briones MJI (1996) A taxonomic revision of the *Allolobophora caliginosa* complex (Oligochaeta, Lumbricidae): a preliminary study. Canadian Journal of Zoology 74: 240–244.

[62] Vickerton P, Jarvis J, Jeffery N (2013) Concentration-dependent specimen shrinkage in iodine-enhanced microCT. Journal of Anatomy 223: 185–193. 23721431 10.1111/joa.12068 PMC3724211

[63] Lenihan J, Kvist S, Fernández R, Giribet G, Ziegler A (2014) MicroCT scans of freshly fixed and museum earthworm specimens. GigaScience Database doi:10.5524/100092 10.1186/2047-217X-3-6 PMC4023164 24839546

[64] Lenihan J, Kvist S, Fernández R, Giribet G, Ziegler A (2014) A dataset comprising four micro-computed tomography scans of freshly fixed and museum earthworm specimens. GigaScience 3: 6. 24839546 10.1186/2047-217X-3-6 PMC4023164

[65] Murienne J, Ziegler A, Ruthensteiner B (2008) A 3D revolution in communicating science. Nature 453: 450. 10.1038/453450d 18497796

[66] Ziegler A, Mietchen D, Faber C, von Hausen W, Schobel C, et al (2011) Effectively incorporating selected multimedia content into medical publications. BMC Medicine 9: 17. 21329532 10.1186/1741-7015-9-17 PMC3040697

[67] Csuzdi C, Pavlicek T, Misirlioglu M (2007) Earthworms (Oligochaeta: Lumbricidae, Criodrilidae and Acanthodrilidae) of Hatay Province, Turkey, with description of three new lumbricids. Acta Zoologica Academiae Scientiarum Hungaricae 53: 347–361.

[68] Csuzdi C, Pop VV (2007) Redescription of *Allolobophora dugesi getica* Pop, 1947 and its allocation to the genus *Cernosvitovia* Omodeo, 1956 (Oligochaeta Lumbricidae). European Journal of Soil Biology 43: S19–S23.

[69] Omodeo P, Rota E (1991) Earthworms of Turkey. II. Bollettino di Zoologia 58: 171–181.

[70] Faraj KA, Cuijpers VMJI, Wismans RG, Walboomers XF, Jansen JA, et al (2009) Micro-computed tomographical imaging of soft biological materials using contrast techniques. Tissue Engineering Part C: Methods 15: 493–499. 19485760 10.1089/ten.tec.2008.0436

[71] Metscher BD (2011) X-ray microtomographic imaging of intact vertebrate embryos. Cold Spring Harbor Protocols 12: 1462–1471. 10.1101/pdb.prot067033 22135670

[72] Pauwels E, Van Loo D, Cornillie P, Brabant L, Van Hoorebeke L (2013) An exploratory study of contrast agents for soft tissue visualization by means of high resolution X-ray computed tomography imaging. Journal of Microscopy 250: 21–31. 23432572 10.1111/jmi.12013

[73] Paredes UM, Prys-Jones R, Adams M, Groombridge J, Kundu S, et al (2012) Micro-CT X-rays do not fragment DNA in preserved bird skins. Journal of Zoological Systematics and Evolutionary Research 50: 247–250.

[74] Ziegler A (2012) Broad application of non-invasive imaging techniques to echinoids and other echinoderm taxa. Zoosymposia 7: 53–70.

