# Supplementary figures and images for: Sine Systemate Chaos? A Versatile Tool for Earthworm Taxonomy: Non-Destructive Imaging of Freshly Fixed and Museum Specimens Using Micro-Computed Tomography
Source: PLoS One. 2014 May 16;9(5):e96617. doi: 10.1371/journal.pone.0096617 (PMC4023944; doi:10.1371/journal.pone.0096617)

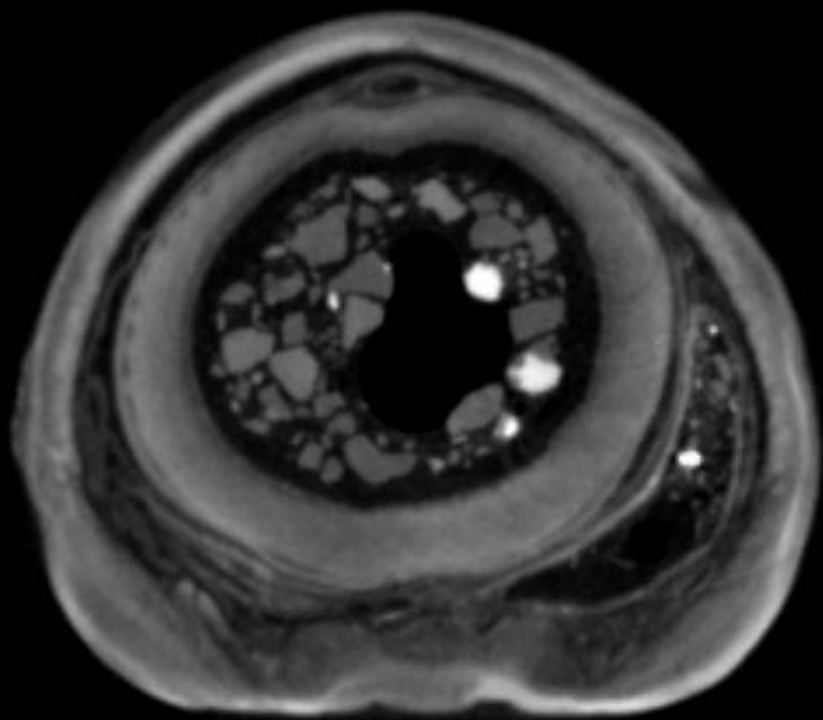

Supplement: File S1 — Video file showing the full image stack of the μCT-scanned, PTA-stained specimen of Aporrectodea caliginosa (MCZ IZ 24805, freshly fixed specimen). (PDF) [file pone.0096617.s001.pdf]

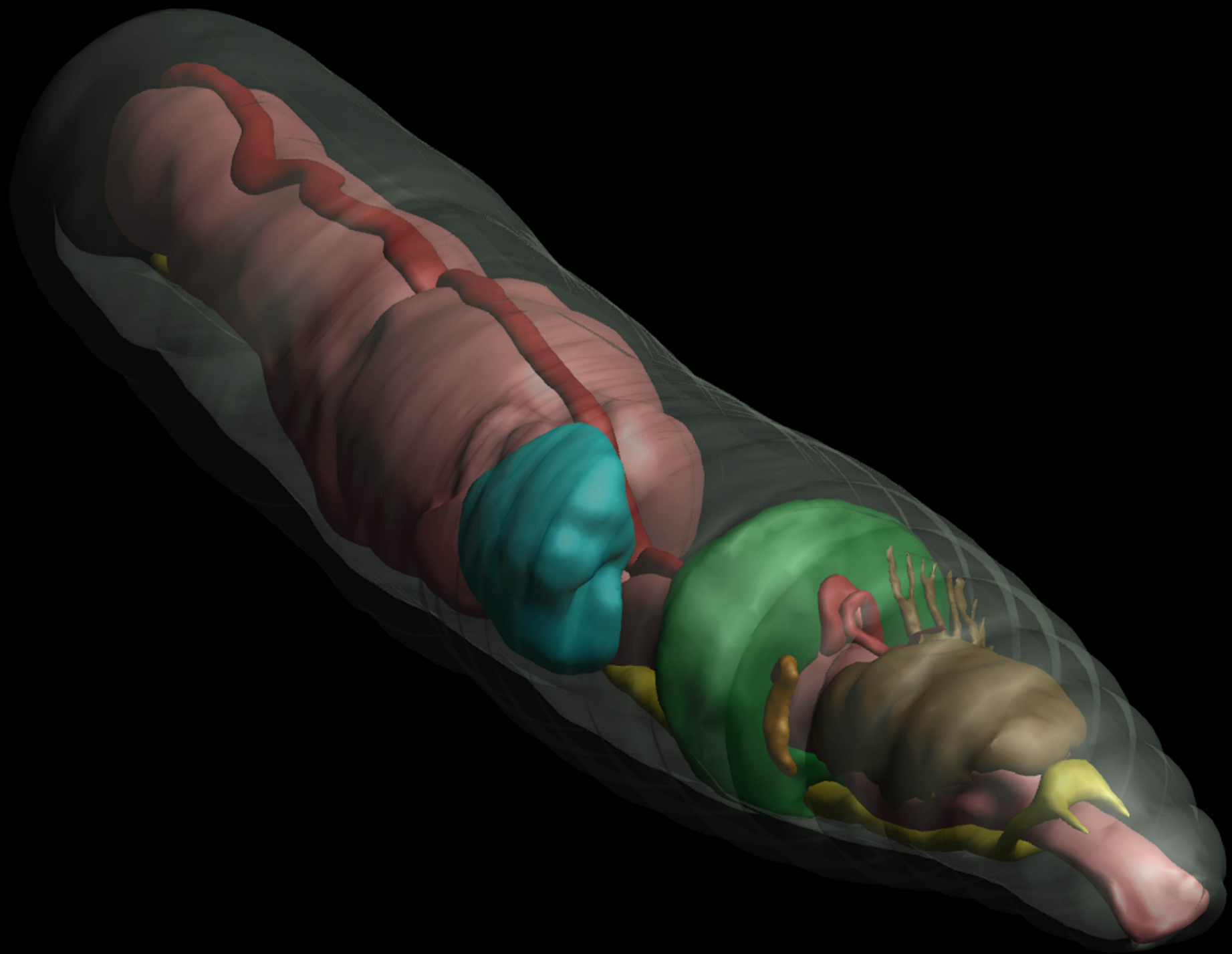

Supplement: File S2 — Interactive 3D PDF model of selected earthworm structures. The 3D model is based on a μCT-scanned, PTA-stained specimen of Aporrectodea caliginosa (MCZ IZ 24805, freshly fixed specimen). Left-click to activate the embedded multimedia content (requires the use of Adobe Reader 9 or higher on Windows, Mac, and Linux systems). Use the ‘+/− zoom’ or ‘toggle full-screen’ options in order to maximize window size. Various pre-saved views can be accessed through the menu inside the viewer window or by opening the model hierarchy using the model tree icon. Grey, body wall; pink, digestive tract; red, circulatory system; brown, pharyngeal musculature; yellow, nervous system; orange, metanephridium; green, muscular septum; cyan, seminal vesicle; blue, spermatheca. (PDF) [file pone.0096617.s002.pdf]

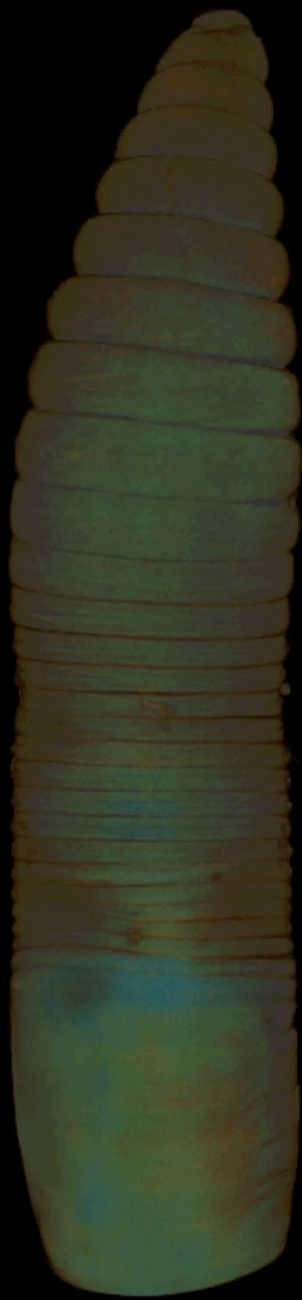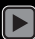

Supplement: File S4 — Video file showing a virtual dissection of the anterior part of a μCT-scanned, PTA-stained specimen of Aporrectodea caliginosa (MCZ IZ 24805, freshly fixed specimen). The video presents volume-rendered, false-colored coronal, sagittal, and transverse 3D views through the body of the specimen. Left-click to activate the embedded multimedia content (requires the use of Adobe Reader 9 or higher on Windows, Mac, and Linux systems). (PDF) [file pone.0096617.s004.pdf]
