# Supplementary material for: Sine Systemate Chaos? A Versatile Tool for Earthworm Taxonomy: Non-Destructive Imaging of Freshly Fixed and Museum Specimens Using Micro-Computed Tomography
Source: PLoS One. 2014 May 16;9(5):e96617. doi: 10.1371/journal.pone.0096617 (PMC4023944; doi:10.1371/journal.pone.0096617)
Supplement: File S3 — Text file providing a step-by-step walkthrough of the pre-saved views shown in the interactive 3D PDF model (File S2). The views mentioned in this description can be accessed through the menu inside the viewer window or by opening the model hierarchy using the model tree icon. (PDF) [file pone.0096617.s003.pdf]

Step-by-step walkthrough of the pre-saved views shown in the interactive 3D PDF model of *A. caliginosa* (Supplemental file 1)

Traditional descriptions based on two-dimensional figures and videos alone can provide only a limited impression of the structural complexity of an organism. Supplemental file 1 provides an embedded 3D model, which permits interactive access to selected internal and external structures of an earthworm in three dimensions. The model is based on the µCT dataset of the PTA-stained specimen of *Aporrectodea caliginosa* (MCZ IZ 24805), described in detail in the Results of the main manuscript. In order not to obfuscate the user's view, structures which are repeated in several segments were reconstructed only in one segment. Furthermore, all structures were smoothed and reduced in their complexity for reasons of file size. The following paragraphs provide a concise walkthrough through the 3D model based on pre-saved views that can be accessed either through the menu in the viewer window or by pressing the model tree icon in the same menu and then switching through the views by pressing the green arrows. However, the 3D model can be manipulated at liberty by the reader at any given point of this walkthrough.

The first view to appear after the activation of the embedded model is the *Standard view*. This view shows the external structure of the organism in antero-dorsal view, revealing segments, intersegmental furrows, and clitellum. By turning the model around, the ventral side is exposed and the male pores can be identified. The following view (*Mouth*) centers on the anterior-most part of the animal as seen from the ventral side. The mouth is located in-between the first two segments, i.e., prostomium and peristomium (segment I). The next view (*Standard view transparent*) provides the same overview as the first view, but the body wall has been rendered transparent in order to reveal all reconstructed internal structures.

The following four views all focus on aspects of digestive tract morphology. The view *Digestive tract w/labels* shows the general shape of the different parts composing the digestive tract (rectum and anus are not part of the scanned field of view). Notable is the significant increase in digestive tract diameter starting with the crop. The views

Pharyngeal musculature and *Pharyngeal musculature transparent* reveal the large muscle located dorsal to the convoluted pharynx. Selected radial muscles have been reconstructed to illustrate their shape and length as well as the location of their attachment sites on the interior side of the body wall. Finally, the view *Crop, gizzard, intestine* centers on the junction between fore- and midgut elements. Of particular interest are the paired, ventral lappets of the crop that project caudally along the proximal part of the gizzard.

One of the muscular septa present in the anterior segments in this group of earthworms is illustrated in the views *Muscular septum I-IV*. The septum can be seen surrounding the ventral nerve cord, the esophagus, and the dorsal blood vessel. Next, the views *Circulatory system I-III* show various aspects of the shape of selected parts of the vascular system. The dorsal blood vessel is in most parts closely associated with the course of the digestive tract. Notable is that the dorsal blood vessel is wound up screw-like in its anterior section. This is likely a response to the contraction of the anterior segments of this freshly fixed, but unrelaxed specimen. The view *Circulatory system IV* reveals the connection of the dorsal blood vessel with the ventral blood vessel (reconstructed only in part) through a large heart. The next set of views, *Nervous system I-IV*, shows a reconstruction of selected nervous tissue elements. While the first three views illustrate their shape and location, the view *Nervous system IV* centers on the anterior part of the nervous system with a paired supraesophageal ganglion, a paired cerebral commissure, and a paired subesophageal ganglion. Interestingly, the ventral cord is - in contrast to the dorsal blood vessel - not wound up in a screw-like fashion in the contracted anterior segments.

The following views (*Metanephridium I-III*) reveal the presence of a J-shaped metanephridium hooking caudally. The proximal part of this excretory structure attaches to a septum (not reconstructed). Finally, the last views (*Reproductive system I-III*) show two exemplary components of the reproductive system of the organism, i.e., one seminal vesicle and one spermatheca.
